# Crystal structures of 2-amino­pyridine citric acid salts: C_5_H_7_N_2_^+^·C_6_H_7_O_7_^−^ and 3C_5_H_7_N_2_^+^·C_6_H_5_O_7_^3−^

**DOI:** 10.1107/S2056989018009787

**Published:** 2018-07-17

**Authors:** Shet M. Prakash, S. Naveen, N. K. Lokanath, P. A. Suchetan, Ismail Warad

**Affiliations:** a Dept. of Chemistry, University College of Science, Tumkur University, Tumkur, 572103, India; b Department of Basic Sciences, School of Engineering and Technology, Jain, University, Bangalore 562 112, India; c Department of Studies in Physics, University of Mysore, Manasagangotri, Mysuru 570 006, India; d Department of Chemistry, Science College, An-Najah National University, PO Box 7, Nablus, Palestinian Territories

**Keywords:** crystal structure, organic salts, 2-amino­pyridinium salts, organic citrates, hydrogen bonding

## Abstract

2-Amino­pyridine and citric acid mixed in 1:1 and 3:1 ratios in ethanol yielded crystals of two 2-amino­pyridinium citrate salts, *viz*. C_5_H_7_N_2_^+^·C_6_H_7_O_7_^−^ (**I**) (systematic name: 2-amino­pyridin-1-ium 3-carb­oxy-2-carb­oxy­methyl-2-hy­droxy­propano­ate), and 3C_5_H_7_N_2_^+^·C_6_H_5_O_7_^3−^ (**II**) [systematic name: tris­(2-amino­pyridin-1-ium) 2-hy­droxy­propane-1,2,3-tri­carboxyl­ate]. The supra­molecular synthons present are analysed and their effect upon the crystal packing is presented in the context of crystal engineering. Salt **I** is formed by the protonation of the pyridine N atom and deprotonation of the central carb­oxy­lic group of citric acid, while in **II** all three carb­oxy­lic groups of the acid are deprotonated and the charges are compensated for by three 2-amino­pyridinium cations. In both structures, a complex supra­molecular three-dimensional architecture is formed. In **I**, the supra­molecular aggregation results from N_amino_—H⋯O_acid_, O_acid_⋯H—O_acid_, O_alcohol_—H⋯O_acid_, N_amino_—H⋯O_alcohol_, N_py_—H⋯O_alcohol_ and C_ar_—H⋯O_acid_ inter­actions. The mol­ecular conformation of the citrate ion (CA^3−^) in **II** is stabilized by an intra­molecular O_alcohol_—H⋯O_acid_ hydrogen bond that encloses an *S*(6) ring motif. The complex three-dimensional structure of **II** features N_amino_—H⋯O_acid_, N_py_—H⋯O_acid_ and several C_ar_—H⋯O_acid_ hydrogen bonds. In the crystal of **I**, the common charge-assisted 2-amino­pyridinium–carboxyl­ate heterosynthon exhibited in many 2-amino­pyridinium carboxyl­ates is not observed, instead chains of N—H⋯O hydrogen bonds and hetero O—H⋯O dimers are formed. In the crystal of **II**, the 2-amino­pyridinium–carboxyl­ate heterosynthon is sustained, while hetero O—H⋯O dimers are not observed. The crystal structures of both salts display a variety of hydrogen bonds as almost all of the hydrogen-bond donors and acceptors present are involved in hydrogen bonding.

## Abstract 2

2-Amino­pyridine and citric acid mixed in 1:1 and 3:1 ratios in ethanol yielded crystals of two 2-amino­pyridine citric acid salts, *viz*. C_5_H_7_N_2_^+^·C_6_H_7_O_7_^−^ (**I**) and 3C_5_H_7_N_2_^+^·C_6_H_5_O_7_^3−^ (**II**). Salt **I** is formed by the protonation of the pyridine N atom and deprotonation of the central carb­oxy­lic group of the acid, while in **II** all three carb­oxy­lic groups of the acid are deprotonated and the charges are compensated for by three 2-amino­pyridinium cations.

## Chemical context   

Systematic structural and statistical analysis focusing on the identification of robust supra­molecular synthons or patterns are essential for crystal engineering and the design of new solid-state structures with desired properties. Organic crystals, especially salts, are now considered as potential materials for optical applications because of their flexibility in mol­ecular design (Jayanalina *et al.*, 2015*a* ▸), thermal stability and delocalized clouds of π electrons (Jayanalina *et al.*, 2015*b* ▸). An analysis of the Cambridge Structural Database (Groom *et al.*, 2016 ▸) by Bis & Zaworotko (2005 ▸) revealed that 77% of compounds that contain both the 2-amino­pyridine and carb­oxy­lic acid moieties generate 2-amino­pyridine–carb­oxy­lic acid supra­molecular heterosynthons rather than carb­oxy­lic acid or 2-amino­pyridine supra­molecular homosynthons. In the absence of other competing functionalities, the occurrence of heterosynthons increased to 97%. Several salts and co-crystals containing 2-amino­pyridine or 2-acetamino­pyridine and a carb­oxy­lic acid moiety have been reported (Jayanalina *et al.*, 2015*a* ▸,*b* ▸; Bis & Zaworotko, 2005 ▸; Aakeröy *et al.*, 2006 ▸; Jasmine *et al.*, 2015 ▸; Jin *et al.*, 2001 ▸). In all of these reported structures, the charge-assisted 2-amino­pyridinium-carboxyl­ate or neutral 2-acetamino­pyridine–carb­oxy­lic heterosynthon is observed, as suggested by statistical analysis. Keeping this in mind, the crystal structure analyses of two 2-amino­pyridinium citrate salts, C_5_H_7_N_2_^+^·C_6_H_7_O_7_^−^ (**I**) and 3C_5_H_7_N_2_^+^·C_6_H_5_O_7_^3−^ (**II**), were undertaken in order to study the packing patterns and identify the supra­molecular synthons present in each salt.
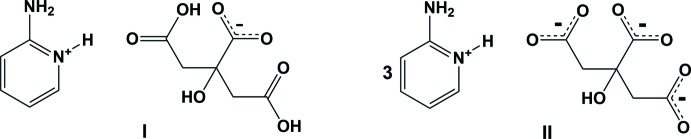


## Structural commentary   

**Figure 1:**
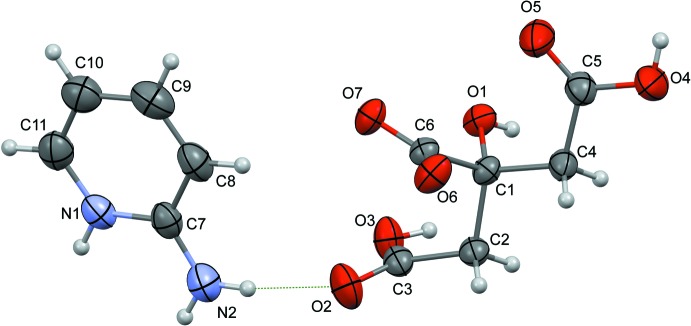
A view of the mol­ecular structure of salt **I**, with the atom labelling. Displacement ellipsoids are drawn at the 50% probability level. Hydrogen bonds are shown as dashed lines [Table 1 ▸; acid(t1) = C3/O2/O3; acid(t2) = C5/O4/O5; acid(c) = C6/O6/O7].

The carb­oxy­lic groups in citric acid have pKa values of 3.128 (central –COOH group), 4.762 and 6.396 (terminal –COOH groups). Thus, an equimolar mixing of citric acid and 2-amino­pyridine resulted in the formation of salt **I** (2-AMP^+^·CA^−^), whose structure is illustrated in Fig. 1 ▸. Here, the pyridine N atom is protonated and the central carb­oxy­lic group of the acid is deprotonated. The two C—O bond lengths of the central carb­oxy­lic group have values of 1.235 (3) Å for C6—O7 and 1.264 (3) Å for C6—O6, indicating partial double-bond character for both bonds. However, the two C—O bonds in each of the terminal carb­oxy­lic groups have different bond lengths [1.207 (3) Å for C3=O2 and 1.327 (3) Å for C3—O3, and 1.209 (3) Å for C5=O5 and 1.319 (3) Å for C5—O4], indicating double-bond character for one C—O bond and single-bond character for the other. These observations clearly confirm the deprotonation of the central carb­oxy­lic group (C6/O6/O7). The two terminal carb­oxy­lic groups in **I** have different conformations. In one of them (C5/O4/O5) the O—H and C=O bonds are in a *syn* conformation while in the other (C3/O2/O3), they have an *anti* conformation (Fig. 1 ▸). In the asymmetric unit of **I**, the 2-amino­pyridinium cation, 2-AMP^+^, and the citrate anion, CA^-^, are linked *via* N_amino_—H⋯O_acid(t1)_ hydrogen bonds [acid(t1) = C3/O2/O3], *viz*. N2—H2*D*⋯O2 (Table 1 ▸ and Fig. 1 ▸).

**Figure 2:**
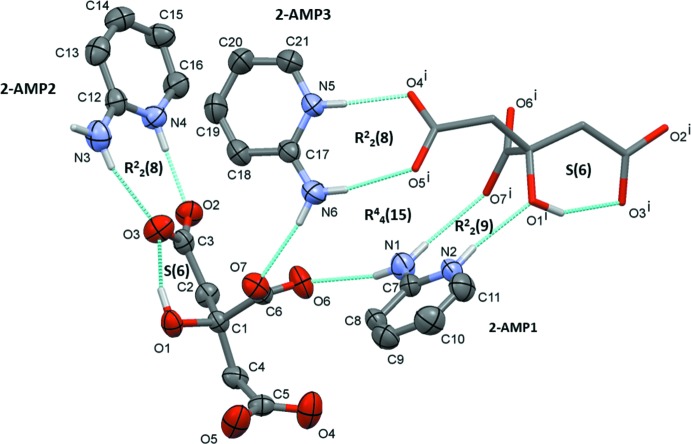
A view of the mol­ecular structure of salt **II**, with the atom labelling. Displacement ellipsoids are drawn at the 50% probability level. Intra­molecular and some inter­molecular inter­actions are shown as dashed lines [Table 2 ▸; acid(t1) = C3/O2/O3; acid (t2) = C5/O4/O5; acid(*c*) = C6/O6/O7; symmetry code: (i) −*x* + 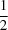, *y* − 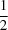, −*z* + 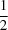]. For clarity, C-bound H atoms have been omitted.

The asymmetric unit of salt **II**, illustrated in Fig. 2 ▸, consists of one citrate trianion, CA^3−^ [(C_5_H_5_O_7_)^3−^], and three 2-AMP^+^ cations (2-AMP1, 2-AMP2 and 2-AMP3), wherein the pyridine N atom of each 2-AMP unit is protonated and all three carb­oxy­lic groups of the acid are deprotonated. This is supported by the observation that the C—O bonds of all the three carb­oxy­lic groups have similar bond lengths, in the range 1.231 (2)–1.266 (2) Å, which is an indication of the partial double-bond character of all of the C—O bonds resulting from deprotonation. The mol­ecular conformation of the CA^3−^ anion is stabilized by an intra­molecular O_alcohol_—H⋯O_acid(t1)_ hydrogen bond, namely O1—H1*O*⋯O3, that closes an *S*(6) ring motif (Table 2 ▸, Fig. 2 ▸).

In the asymmetric unit of salt **II**, the three 2-AMP^+^ cations are in different environments and inter­act with the CA^3−^ anion in different ways [Fig. 2 ▸ and Table 2 ▸; acid(t1) = C3/O2/O3; acid(t2) = C5/O4/O5; acid(c) = C6/O6/O7]. The first cation, 2-AMP1, inter­acts with the anion *via* a discrete N_amino_—H⋯O_acid(c)_ hydrogen bond, namely N1—H1*B*⋯O6. The second cation, 2-AMP2, inter­acts with the CA^3−^ anion *via* a charge-assisted 2-amino­pyridinium-carboxyl­ate 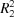
(8) heterosynthon consisting of N_amino_—H⋯O_acid(t1)_ (N3—H3*A*⋯O3) and N_py_—H⋯O_acid(t1)_ (N4—H4⋯O2) hydrogen bonds. The third cation, 2-AMP3, inter­acts with the anion *via* a discrete N_amino_—H⋯O_acid(c)_ hydrogen bond, namely N6—H6*B*⋯O7.

## Supra­molecular features   

**Figure 3:**
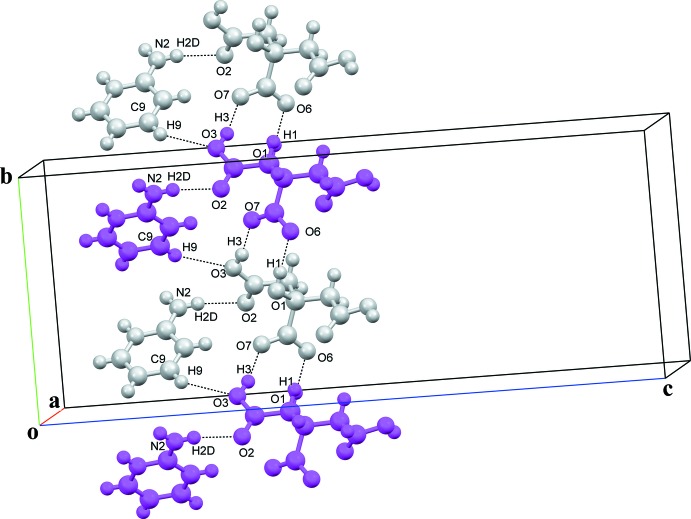
A partial view along the *a* axis of the crystal packing of salt **I**, showing the chains of CA^−^ anions running along the *b*-axis direction. Attached to the chains and bridging two anions are the 2-AMP^+^ cations. The various inter­molecular inter­actions are shown as dashed lines (Table 1 ▸).

**Figure 4:**
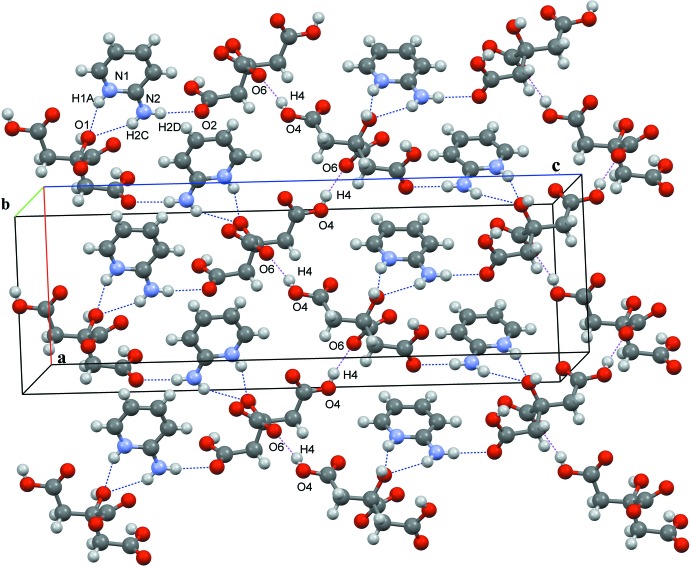
A partial view along the *b* axis of the crystal packing of salt **I**, illustrating the layer-like structure. Red and blue dashed lines denote the various inter­molecular inter­actions (Table 1 ▸).

Full details of the hydrogen-bonding inter­actions in the crystal of salt **I** are given in Table 1 ▸, and illustrated in Figs. 3 ▸ and 4 ▸. In the crystal of **I**, the cations and anions of adjacent units are inter­connected by a C_ar_—H⋯O_acid(t1)_ inter­actions, *viz*. C9—H9⋯O3, while adjacent anions related by *b*-glide symmetry form chains running along the *b-*axis direction, consisting of an 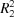
(8) heterosynthon of O_acid(c)_⋯H—O_acid(t1)_ and O_alcohol_—H⋯O_acid(c)_ hydrogen bonds, namely O3—H3⋯O7^i^ and O1—H1⋯O6^i^; see Fig. 3 ▸ and Table 1 ▸. The 2-AMP^+^ and CA^−^ ions further aggregate to form sheets parallel to the *ac* plane (Fig. 4 ▸). The sheets consist of chains of O_acid(t2)_—H⋯O_acid(c)_ hydrogen bonds, namely O4—H4⋯O6^iii^, running along the *a-*axis direction and linking the twofold-symmetry-related CA^−^ anions (Table 1 ▸, Fig. 4 ▸). Adjacent chains are connected by 2-AMP^+^ ions *via* N_amino_—H⋯O_acid(t1)_=C hydrogen bonds, namely N2—H2*D*⋯O2, and an 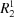
(6) heterosynthon of N_amino_—H⋯O_alcohol_ and N_py_—H⋯O_alcohol_ hydrogen bonds, N2—H2*C*⋯O1^ii^ and N1—H1*A*⋯O1^ii^, respectively, is formed (Table 1 ▸, Fig. 4 ▸). Overall, a three-dimensional supra­molecular architecture is observed. All of the strong hydrogen-bond acceptors and hydrogen-bond donors in **I** are involved in hydrogen bonding. However, the most reproducible charge-assisted 2-amino­pyridinium–carboxyl­ate heterosynthon, found in the crystal structures of many 2-amino­pyridinium carboxyl­ates (Bis & Zaworotko, 2005 ▸), is not present; instead chains of N—H⋯O hydrogen bonds and hetero O—H⋯O dimers are observed.

**Figure 5:**
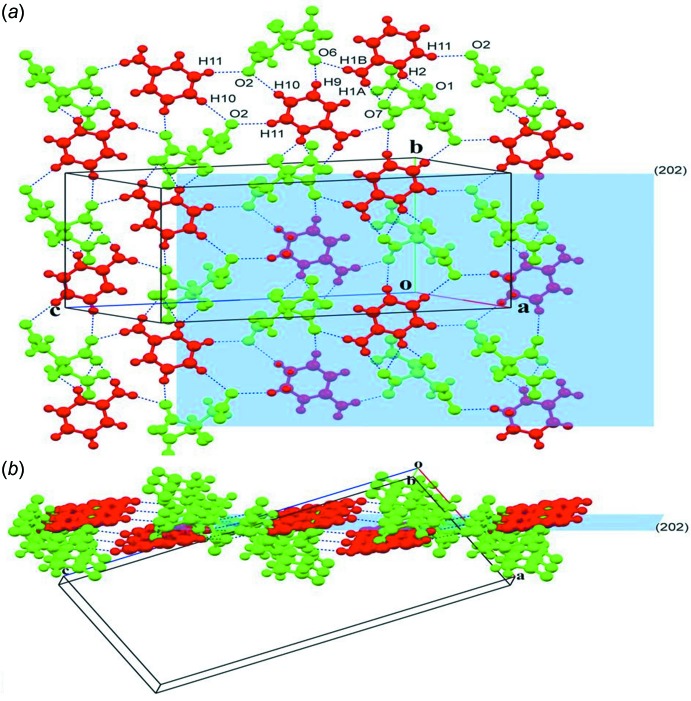
(*a*) Partial crystal packing of salt **II**, involving citrate (green) and 2-AMP1 (red) ions, showing the layer-like structure lying in plane (202). (*b*) An alternative view, along the *b* axis, of the layer-like structure. The hydrogen bonds and other inter­molecular inter­actions are shown as dashed lines (Table 2 ▸).

**Figure 6:**
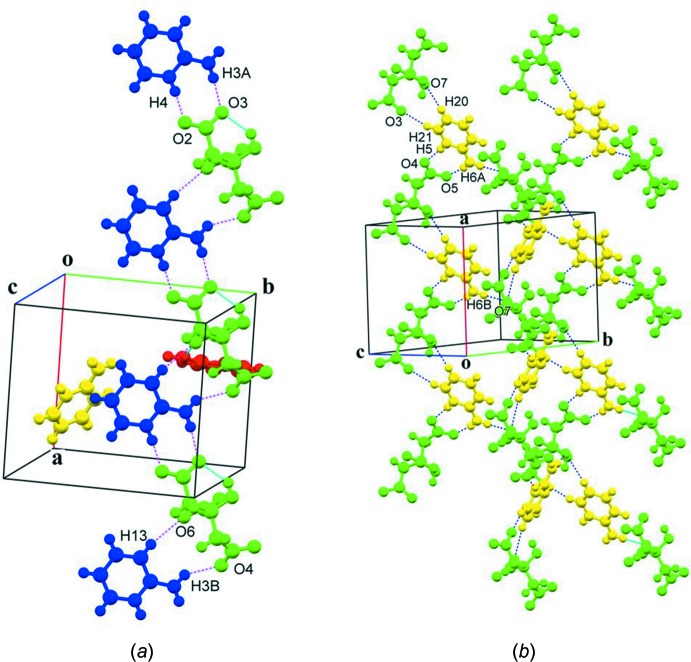
(*a*) Partial crystal packing of salt **II**, involving citrate (green) and 2-AMP2 (blue) ions. Red dashed lines denote various inter­molecular inter­actions and solid blue lines denote intra­molecular hydrogen bonds (Table 2 ▸). (*b*) Partial crystal packing of salt **II**, involving citrate (green) and 2-AMP3 (yellow) ions. Dashed lines denote various inter­molecular inter­actions (Table 2 ▸).

In the crystal of **II**, all of the strong hydrogen-bond donors and acceptors are utilized in a supra­molecular association. Full details of the hydrogen-bonding inter­actions are given in Table 2 ▸, and illustrated in Figs. 2 ▸, 5 ▸ and 6 ▸. A number of the C_ar_—H groups are also involved in C—H⋯O hydrogen bonds (Table 2 ▸). However, in contrast to **I**, the alcoholic OH group is not involved in inter­molecular hydrogen bonding as it is locked into an intra­molecular O1—H1*O*⋯O3_acid(t1)_ hydrogen bond. The CA^3−^ anion and the first 2-AMP^+^ cation (2-AMP1) form sheets lying parallel to the (101) plane (Fig. 5 ▸ *a* and 5*b*). The sheet consists of alternating CA^3−^ and 2-AMP^+^ ions, forming chains *via* C11—H11⋯O2^iii^ inter­actions, with adjacent anti-parallel chains linked by C10—H10⋯O2^ii^, N1—H1*A*⋯O7^i^, N1—H1*B*⋯O6, N2—H2⋯O7^i^ and N2—H2⋯O1^i^ hydrogen bonds (Table 2 ▸, Fig. 5 ▸). On the other hand, the citrate and the second 2-AMP^+^ ions (2-AMP2) propagate alternately along the *a*-axis direction to form ribbons (Fig. 6 ▸ *a*) consisting of alternating 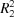
(8) heterosynthons of N3—H3*A*⋯O3 and N4—H4⋯O2 hydrogen bonds (Table 2 ▸) and 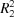
(11) heterosynthons of N3—H3*B*⋯O4 and C13—H13⋯O6 hydrogen bonds (Table 2 ▸). Finally, the third 2-AMP^+^ ions (2-AMP3) are inter­linked to the adjacent citrate ions, forming ribbons of alternating 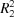
(8) heterosynthons, of N5—H5⋯O4^i^ and N6—H6*A*⋯O5^i^ hydrogen bonds (Table 2 ▸), and 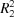
(10) heterosynthons of C21—H21⋯O3^vi^ and C20—H20⋯O7^vi^ inter­actions (Table 2 ▸) along the *a*-axis direction (Fig. 6 ▸ *b*). Adjacent ribbons are further inter­connected by N6—H6*B*⋯O7 hydrogen bonds to form corrugated sheets parallel to the *ab* plane (Table 2 ▸, Fig. 6 ▸ *b*). Overall a complex supra­molecular three-dimensional structure is formed.

## Database survey   

A survey of the Cambridge Structural Database (CSD, Version 5.39, last update May 2018; Groom *et al.*, 2016 ▸) revealed 80 organic structures involving a citric acid moiety in the form of solvates/hydrates, salts/salt hydrates and co-crystals. 25 structures among these are salts/salt hydrates of citric acid (deprotonated to different extents) with various organic cations. It is observed that most of the organic citrates appear as their hydrates, with the exception of a few (including **I** and **II**). The most common hydrogen bonds observed in these hydrated salts are N_amine_—H⋯O_citric_, N_amine_—H⋯O_water_ and O_water_—H⋯O_citric_, forming different supra­molecular architectures. In the absence of a water mol­ecule, the most common hydrogen bonds are N_amine_—H⋯O_citric_ and O_citric_—H⋯O_citric_. However, the nature of these supra­molecular synthons varies from one structure to another, depending on the nature of the organic cations.

Similarly, the crystal structures of several salts with 2-AMP^+^ as the cation are reported. Single-crystal structures of ten salts that contain both a 2-amino­pyridine and a carb­oxy­lic acid moiety have been reported (Bis & Zaworotko, 2005 ▸). They include: 2-amino­pyridinium 4-amino­benzoate, 2-amino­pyridinium isophthalate, bis­(2-amino­pyridinium) terephthal­ate, 2-amino-5-methyl­pyridinium benzoate, bis­(2-amino-5-methyl­pyridinium) 5-tertbutyl­isophthalate, 2-amino-5-meth­yl­pyridinium terephthalate, bis­(2-amino-5-methyl­pyridinium) 2,6-naphthalenedi­carboxyl­ate, bis­(2-amino 5-methyl­pyrid­in­ium) adipate adipic acid, bis­(2-amino-5-methyl­pyridinium) 2,5-thio­phenedi­carboxyl­ate 2,5-thio­phenedi­carb­oxy­lic acid, and indomethacin 2-amino-5-methyl­pyridinium. In all the reported structures, the most reproducible pattern is the charge-assisted 2-amino­pyridinium–carboxyl­ate heterosynthon seen in salt **II**. Similarly, in the crystal structure of 2-amino-3-methyl­pyridinium *ortho*-phthalate (Jin *et al.*, 2001 ▸), the two 2-amino-3-methyl­pyridinium ions are inter­connected to the *ortho*-phthalate ion *via* a charge-assisted 2-amino­pyridinium–carboxyl­ate heterosynthon. This robust pattern is also observed in the crystal structures of 2-amino­pyridinium 6-chloro­nicotinate (Jasmine *et al.*, 2015 ▸) and 2-amino-5-chloro­pyridinium pyridine-2-carboxyl­ate monohydrate (Jayanalina *et al.*, 2015*a* ▸). Single-crystal structures of ten co-crystals that contain 2-acetamino­pyridine and a carb­oxy­lic acid moiety: 2-acetamino­pyridine/fumaric acid have been reported by Aakeröy *et al.* (2006 ▸). They include: 2-acetamino­pyridine/succinic acid, 2-acetamino­pyridine/glutaric acid, 2-acet­amino­pyridine /adipic acid, 2-acetamino­pyridine/pimelic acid, 2-acetamino­pyridine/suberic acid, 2-acetamino-pyridine/azelaic acid, 2-acetamino­pyridine/sebacic acid, 2-acetamino­pyridine/3,5-di­methyl­benzoic acid, and 2-acetamino­pyridine/5-nitro­isophthalic acid. Although these are neutral compounds wherein there is no transfer of proton from carb­oxy­lic acid to the 2-acetamino­pyridine moiety, the most repetitive pattern observed in these structures is the neutral 2-acetamino­pyridine–carb­oxy­lic acid 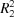
(8) heterosynthon. This is very similar to the charge-assisted 2-amino­pyridinium–carboxyl­ate heterosynthon except for the positioning of the hydrogen atom, on either the O or N atom.

The crystal structure of 2-amino 5-chloro­pyridinium-l-tartarate (Jayanalina *et al.*, 2015*b* ▸) shows that despite of the presence of other competing functionalities on the carb­oxy­lic acid (two alcoholic OH groups in tartaric acid), the most frequent 2-amino­pyridinium–carboxyl­ate heterosynthon is still observed. However, the presence of the alcoholic OH group in citric acid has resulted in a deviation from the regular trend as the charge-assisted 2-amino­pyridinium–carboxyl­ate heterosynthon is not observed in **I**; instead chains of N—H⋯O hydrogen bonds and hetero O—H⋯O dimers are observed. The 2-amino­pyridinium–carboxyl­ate heterosynthon is sustained in the crystal structure of **II** because of the non-availability of the alcoholic OH group for inter­molecular hydrogen bonding.

Hence, the study of the crystal structure of 2-amino­pyridinium citrate, mixed in a 2:1 ratio, would be highly significant in understanding the packing-pattern trends observed in this family of salts. Unfortunately, despite a number of attempts, we have not been able to obtain good-quality single crystals of this salt.

## Synthesis and crystallization   

A solution of citric acid (3 mmol, 0.576 g) in ethanol (15 ml) was added to an ethano­lic solution (15 ml) of 2-amino­pyridine (3 mmol, 0.282 g). The resulting solution was heated and the hot solution was filtered. Slow evaporation of the solution resulted in the formation of colourless prismatic crystals of salt **I** (m.p. 493 K). Single crystals of salt **II** were obtained from a similar procedure; an ethano­lic solution (15 ml) of citric acid (3 mmol, 0.576 g) was mixed with an ethano­lic solution (15 ml) of 2-amino­pyridine (9 mmol, 0.846 g).

## Refinement details   

Crystal data, data collection and structure refinement details are summarized in Table 3 ▸. In salt **I**, the OH H atom (H1) was positioned geometrically and refined as riding: O—H = 0.82 Å with *U*_iso_(H) = 1.5*U*_eq_(O). In salt **II**, the OH H atom (H1*O*) was located in a difference-Fourier map and freely refined. In both salts, the other H atoms were positioned geometrically and refined as riding: N—H = 0.86 Å, C—H = 0.93–0.97 Å with *U*_iso_(H) = 1.2*U*_eq_(N, C).

## Supplementary Material

Crystal structure: contains datablock(s) I, II, Global. DOI: 10.1107/S2056989018009787/su5449sup1.cif

Structure factors: contains datablock(s) I. DOI: 10.1107/S2056989018009787/su5449Isup2.hkl

Structure factors: contains datablock(s) II. DOI: 10.1107/S2056989018009787/su5449IIsup3.hkl

Supporting information file. DOI: 10.1107/S2056989018009787/su5449Isup4.cml

Supporting information file. DOI: 10.1107/S2056989018009787/su5449IIsup5.cml

CCDC references: 1854628, 1854627

Additional supporting information:  crystallographic information; 3D view; checkCIF report

## Figures and Tables

**Table 1:** Hydrogen-bond geometry (Å, °) for **I** [Chem scheme1]

*D*—H⋯*A*	*D*—H	H⋯*A*	*D*⋯*A*	*D*—H⋯*A*
O1—H1⋯O6^i^	0.82	1.86	2.681 (4)	177
N1—H1*A*⋯O1^ii^	0.86	2.09	2.895 (4)	156
N2—H2*C*⋯O1^ii^	0.86	2.34	3.076 (5)	144
N2—H2*D*⋯O2	0.86	2.09	2.935 (5)	168
O3—H3⋯O7^i^	0.82	1.75	2.547 (4)	164
O4—H4⋯O6^iii^	0.82	1.82	2.601 (4)	158
C9—H9⋯O3^iv^	0.93	2.57	3.351 (5)	142

Symmetry codes: (i) 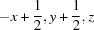; (ii) 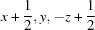; (iii) 
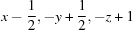
; (iv) 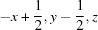.

**Table 2:** Hydrogen-bond geometry (Å, °) for **II** [Chem scheme1]

*D*—H⋯*A*	*D*—H	H⋯*A*	*D*⋯*A*	*D*—H⋯*A*
O1—H1*O*⋯O3	0.91 (3)	1.84 (3)	2.681 (2)	152 (3)
N3—H3*A*⋯O3	0.86	2.07	2.905 (3)	164
N4—H4⋯O2	0.86	1.81	2.666 (2)	175
N1—H1*B*⋯O6	0.86	2.07	2.893 (2)	161
N6—H6*B*⋯O7	0.86	2.09	2.928 (2)	164
N1—H1*A*⋯O7^i^	0.86	2.12	2.948 (2)	162
N2—H2⋯O1^i^	0.86	2.00	2.760 (2)	144
N2—H2⋯O7^i^	0.86	2.55	3.304 (2)	144
C9—H9⋯O6^ii^	0.93	2.60	3.372 (3)	141
C10—H10⋯O2^ii^	0.93	2.51	3.419 (3)	167
C11—H11⋯O2^iii^	0.93	2.41	3.294 (3)	160
N3—H3*B*⋯O4^iv^	0.86	2.09	2.851 (2)	146
C13—H13⋯O6^iv^	0.93	2.40	3.301 (3)	163
N5—H5⋯O4^i^	0.86	1.77	2.591 (2)	160
N6—H6*A*⋯O5^i^	0.86	2.07	2.916 (3)	169
C20—H20⋯O7^v^	0.93	2.60	3.463 (3)	155
C21—H21⋯O3^v^	0.93	2.43	3.334 (3)	164

Symmetry codes: (i) 
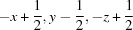
; (ii) 
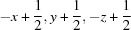
; (iii) 
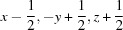
; (iv) 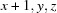; (v) 
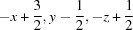
.

**Table 3:** Experimental details

	**I**	**II**
Crystal data		
Chemical formula	C_5_H_7_N_2_^+^·C_6_H_7_O_7_^−^	3C_5_H_7_N_2_^+^·C_6_H_5_O_7_^3−^
*M*_r_	286.24	474.48
Crystal system, space group	Orthorhombic, *P* *b* *c* *a*	Monoclinic, *P*2_1_/*n*
Temperature (K)	296	296
*a*, *b*, *c* (Å)	9.000 (11), 10.721 (13), 27.21 (3)	10.0297 (17), 10.6564 (14), 21.986 (4)
α, β, γ (°)	90, 90, 90	90, 101.426 (9), 90
*V* (Å^3^)	2625 (5)	2303.3 (7)
*Z*	8	4
Radiation type	Mo *K*α	Mo *K*α
μ (mm^−1^)	0.12	0.11
Crystal size (mm)	0.27 × 0.22 × 0.19	0.22 × 0.19 × 0.17

Data collection		
Diffractometer	Bruker APEXII	Bruker APEXII
Absorption correction	Multi-scan (*SADABS*; Bruker, 2009 ▸)	Multi-scan (*SADABS*; Bruker, 2009 ▸)
*T*_min_, *T*_max_	0.968, 0.977	0.977, 0.982
No. of measured, independent and observed [*I* > 2σ(*I*)] reflections	8086, 2977, 2143	13120, 5242, 3779
*R*_int_	0.099	0.056
(sin θ/λ)_max_ (Å^−1^)	0.649	0.650

Refinement		
*R*[*F* ^2^ > 2σ(*F* ^2^)], *wR*(*F* ^2^), *S*	0.066, 0.195, 1.06	0.052, 0.149, 1.05
No. of reflections	2977	5242
No. of parameters	184	311
H-atom treatment	H-atom parameters constrained	H atoms treated by a mixture of independent and constrained refinement
Δρ_max_, Δρ_min_ (e Å^−3^)	0.29, −0.30	0.27, −0.21

Computer programs: *APEX2*, *SAINT-Plus* and *XPREP* (Bruker, 2009 ▸), *SHELXT2016* (Sheldrick, 2015*a* ▸), *SHELXL2016* (Sheldrick, 2015*b* ▸) and *Mercury* (Macrae *et al.*, 2008 ▸).

## supplementary crystallographic information

### 2-Aminopyridin-1-ium 3-carboxy-2-carboxymethyl-2-hydroxypropanoate (I) Crystal data

**Table:**

C_5_H_7_N_2_^+^·C_6_H_7_O_7_^−^	*D*_x_ = 1.448 Mg m^−^^3^
*M**_r_* = 286.24	Melting point: 493 K
Orthorhombic, *P**b**c**a*	Mo *K*α radiation, λ = 0.71073 Å
Hall symbol: -P 2ac 2ab	Cell parameters from 143 reflections
*a* = 9.000 (11) Å	θ = 3.1–27.5°
*b* = 10.721 (13) Å	µ = 0.12 mm^−^^1^
*c* = 27.21 (3) Å	*T* = 296 K
*V* = 2625 (5) Å^3^	Prism, colourless
*Z* = 8	0.27 × 0.22 × 0.19 mm
*F*(000) = 1200	

### 2-Aminopyridin-1-ium 3-carboxy-2-carboxymethyl-2-hydroxypropanoate (I) Data collection

**Table:**

Bruker APEXII diffractometer	2977 independent reflections
Radiation source: sealed X-ray tube	2143 reflections with *I* > 2σ(*I*)
Graphite monochromator	*R*_int_ = 0.099
phi and φ scans	θ_max_ = 27.5°, θ_min_ = 3.1°
Absorption correction: multi-scan (SADABS; Bruker, 2009)	*h* = −11→9
*T*_min_ = 0.968, *T*_max_ = 0.977	*k* = −13→13
8086 measured reflections	*l* = −12→35

### 2-Aminopyridin-1-ium 3-carboxy-2-carboxymethyl-2-hydroxypropanoate (I) Refinement

**Table:**

Refinement on *F*^2^	Primary atom site location: structure-invariant direct methods
Least-squares matrix: full	Secondary atom site location: difference Fourier map
*R*[*F*^2^ > 2σ(*F*^2^)] = 0.066	Hydrogen site location: inferred from neighbouring sites
*wR*(*F*^2^) = 0.195	H-atom parameters constrained
*S* = 1.06	*w* = 1/[σ^2^(*F*_o_^2^) + (0.1065*P*)^2^] where *P* = (*F*_o_^2^ + 2*F*_c_^2^)/3
2977 reflections	(Δ/σ)_max_ < 0.001
184 parameters	Δρ_max_ = 0.29 e Å^−^^3^
0 restraints	Δρ_min_ = −0.30 e Å^−^^3^

### 2-Aminopyridin-1-ium 3-carboxy-2-carboxymethyl-2-hydroxypropanoate (I) Special details

**Table:**

Geometry. All esds (except the esd in the dihedral angle between two l.s. planes) are estimated using the full covariance matrix. The cell esds are taken into account individually in the estimation of esds in distances, angles and torsion angles; correlations between esds in cell parameters are only used when they are defined by crystal symmetry. An approximate (isotropic) treatment of cell esds is used for estimating esds involving l.s. planes.

### 2-Aminopyridin-1-ium 3-carboxy-2-carboxymethyl-2-hydroxypropanoate (I) Fractional atomic coordinates and isotropic or equivalent isotropic displacement parameters (Å^2^)

**Table:**

	*x*	*y*	*z*	*U*_iso_*/*U*_eq_	
C1	0.2827 (2)	0.40538 (18)	0.41150 (7)	0.0284 (4)	
C2	0.4265 (2)	0.47554 (18)	0.39605 (8)	0.0314 (5)	
H2A	0.419854	0.561864	0.406495	0.038*	
H2B	0.511292	0.438155	0.412406	0.038*	
C3	0.4500 (2)	0.47082 (18)	0.34102 (8)	0.0329 (5)	
C4	0.2668 (3)	0.4112 (2)	0.46729 (8)	0.0348 (5)	
H4A	0.354470	0.374637	0.482187	0.042*	
H4B	0.262245	0.497923	0.477316	0.042*	
C5	0.1315 (3)	0.3448 (2)	0.48646 (8)	0.0383 (5)	
C6	0.2956 (2)	0.26921 (17)	0.39304 (7)	0.0280 (4)	
C7	0.4276 (3)	0.3173 (2)	0.19589 (9)	0.0397 (5)	
C8	0.3305 (3)	0.2475 (2)	0.22619 (9)	0.0479 (6)	
H8	0.333716	0.255755	0.260195	0.057*	
C9	0.2320 (3)	0.1677 (3)	0.20452 (11)	0.0546 (7)	
H9	0.167956	0.121120	0.224075	0.066*	
C10	0.2259 (3)	0.1548 (3)	0.15313 (10)	0.0523 (7)	
H10	0.158588	0.100241	0.138646	0.063*	
C11	0.3192 (3)	0.2228 (2)	0.12526 (10)	0.0469 (6)	
H11	0.316651	0.215571	0.091216	0.056*	
N1	0.4170 (2)	0.30207 (19)	0.14690 (7)	0.0411 (5)	
H1A	0.475232	0.344774	0.128331	0.049*	
N2	0.5275 (2)	0.3966 (2)	0.21382 (8)	0.0521 (5)	
H2C	0.584424	0.437389	0.194183	0.062*	
H2D	0.535185	0.406964	0.245054	0.062*	
O1	0.15695 (16)	0.45874 (12)	0.38791 (6)	0.0337 (4)	
H1	0.147677	0.531534	0.396642	0.051*	
O2	0.5321 (2)	0.39665 (16)	0.32168 (7)	0.0520 (5)	
O3	0.3748 (2)	0.55002 (14)	0.31300 (6)	0.0444 (4)	
H3	0.334824	0.602731	0.330373	0.067*	
O4	0.1087 (2)	0.3705 (2)	0.53325 (7)	0.0570 (5)	
H4	0.031941	0.336621	0.542459	0.085*	
O5	0.0527 (2)	0.27613 (19)	0.46272 (7)	0.0620 (6)	
O6	0.37012 (18)	0.19472 (13)	0.41930 (6)	0.0405 (4)	
O7	0.23360 (18)	0.24341 (13)	0.35387 (5)	0.0367 (4)	

### 2-Aminopyridin-1-ium 3-carboxy-2-carboxymethyl-2-hydroxypropanoate (I) Atomic displacement parameters (Å^2^)

**Table:**

	*U*^11^	*U*^22^	*U*^33^	*U*^12^	*U*^13^	*U*^23^
C1	0.0314 (10)	0.0247 (9)	0.0289 (10)	−0.0017 (8)	−0.0027 (8)	−0.0002 (7)
C2	0.0336 (10)	0.0277 (9)	0.0330 (11)	−0.0039 (8)	0.0026 (9)	−0.0010 (8)
C3	0.0370 (11)	0.0259 (9)	0.0357 (11)	−0.0037 (8)	0.0063 (9)	−0.0014 (8)
C4	0.0407 (11)	0.0327 (11)	0.0311 (11)	−0.0065 (9)	0.0047 (9)	−0.0054 (8)
C5	0.0435 (12)	0.0366 (11)	0.0349 (11)	−0.0043 (10)	0.0063 (10)	0.0000 (9)
C6	0.0301 (9)	0.0228 (9)	0.0311 (10)	−0.0014 (7)	−0.0007 (8)	0.0008 (7)
C7	0.0381 (11)	0.0425 (12)	0.0385 (12)	0.0097 (10)	0.0049 (10)	−0.0016 (9)
C8	0.0482 (13)	0.0588 (15)	0.0366 (13)	0.0082 (11)	0.0096 (11)	0.0059 (11)
C9	0.0454 (14)	0.0573 (16)	0.0611 (17)	0.0027 (12)	0.0126 (13)	0.0104 (13)
C10	0.0455 (14)	0.0552 (15)	0.0563 (16)	0.0027 (12)	0.0013 (12)	0.0001 (12)
C11	0.0443 (13)	0.0553 (14)	0.0412 (13)	0.0107 (12)	−0.0018 (11)	−0.0019 (11)
N1	0.0379 (10)	0.0478 (11)	0.0375 (11)	0.0055 (9)	0.0070 (8)	0.0048 (8)
N2	0.0530 (12)	0.0609 (13)	0.0422 (11)	−0.0033 (11)	0.0095 (10)	−0.0061 (10)
O1	0.0339 (8)	0.0232 (7)	0.0441 (9)	0.0033 (6)	−0.0046 (6)	−0.0015 (6)
O2	0.0625 (11)	0.0472 (10)	0.0462 (10)	0.0155 (9)	0.0142 (9)	−0.0037 (8)
O3	0.0641 (11)	0.0364 (9)	0.0326 (8)	0.0135 (8)	0.0050 (8)	0.0007 (6)
O4	0.0542 (10)	0.0787 (13)	0.0381 (10)	−0.0225 (10)	0.0135 (8)	−0.0070 (9)
O5	0.0683 (12)	0.0697 (12)	0.0481 (11)	−0.0336 (11)	0.0144 (10)	−0.0148 (9)
O6	0.0507 (9)	0.0252 (7)	0.0456 (9)	0.0038 (7)	−0.0156 (7)	0.0019 (6)
O7	0.0470 (9)	0.0276 (8)	0.0355 (8)	0.0002 (6)	−0.0087 (7)	−0.0036 (6)

### 2-Aminopyridin-1-ium 3-carboxy-2-carboxymethyl-2-hydroxypropanoate (I) Geometric parameters (Å, º)

**Table:**

C1—O1	1.421 (3)	C7—N1	1.346 (3)
C1—C4	1.526 (3)	C7—C8	1.416 (4)
C1—C6	1.548 (3)	C8—C9	1.365 (4)
C1—C2	1.555 (3)	C8—H8	0.9300
C2—C3	1.513 (3)	C9—C10	1.406 (4)
C2—H2A	0.9700	C9—H9	0.9300
C2—H2B	0.9700	C10—C11	1.346 (4)
C3—O2	1.207 (3)	C10—H10	0.9300
C3—O3	1.327 (3)	C11—N1	1.357 (3)
C4—C5	1.503 (3)	C11—H11	0.9300
C4—H4A	0.9700	N1—H1A	0.8600
C4—H4B	0.9700	N2—H2C	0.8600
C5—O5	1.209 (3)	N2—H2D	0.8600
C5—O4	1.319 (3)	O1—H1	0.8200
C6—O7	1.235 (3)	O3—H3	0.8200
C6—O6	1.264 (3)	O4—H4	0.8200
C7—N2	1.330 (3)		
			
O1—C1—C4	110.98 (18)	O6—C6—C1	116.87 (18)
O1—C1—C6	107.03 (16)	N2—C7—N1	119.3 (2)
C4—C1—C6	111.61 (16)	N2—C7—C8	122.8 (2)
O1—C1—C2	110.23 (17)	N1—C7—C8	117.9 (2)
C4—C1—C2	109.12 (17)	C9—C8—C7	118.7 (3)
C6—C1—C2	107.80 (17)	C9—C8—H8	120.6
C3—C2—C1	111.57 (17)	C7—C8—H8	120.6
C3—C2—H2A	109.3	C8—C9—C10	121.1 (3)
C1—C2—H2A	109.3	C8—C9—H9	119.5
C3—C2—H2B	109.3	C10—C9—H9	119.5
C1—C2—H2B	109.3	C11—C10—C9	118.9 (3)
H2A—C2—H2B	108.0	C11—C10—H10	120.6
O2—C3—O3	118.9 (2)	C9—C10—H10	120.6
O2—C3—C2	122.6 (2)	C10—C11—N1	119.9 (3)
O3—C3—C2	118.45 (18)	C10—C11—H11	120.0
C5—C4—C1	113.69 (18)	N1—C11—H11	120.0
C5—C4—H4A	108.8	C7—N1—C11	123.5 (2)
C1—C4—H4A	108.8	C7—N1—H1A	118.3
C5—C4—H4B	108.8	C11—N1—H1A	118.3
C1—C4—H4B	108.8	C7—N2—H2C	120.0
H4A—C4—H4B	107.7	C7—N2—H2D	120.0
O5—C5—O4	123.5 (2)	H2C—N2—H2D	120.0
O5—C5—C4	125.3 (2)	C1—O1—H1	109.5
O4—C5—C4	111.2 (2)	C3—O3—H3	109.5
O7—C6—O6	125.90 (19)	C5—O4—H4	109.5
O7—C6—C1	117.22 (17)		
			
O1—C1—C2—C3	−59.0 (2)	C2—C1—C6—O7	−97.6 (2)
C4—C1—C2—C3	178.89 (16)	O1—C1—C6—O6	−159.64 (18)
C6—C1—C2—C3	57.5 (2)	C4—C1—C6—O6	−38.0 (3)
C1—C2—C3—O2	−98.1 (3)	C2—C1—C6—O6	81.8 (2)
C1—C2—C3—O3	80.5 (2)	N2—C7—C8—C9	−179.6 (2)
O1—C1—C4—C5	58.9 (2)	N1—C7—C8—C9	0.5 (3)
C6—C1—C4—C5	−60.4 (2)	C7—C8—C9—C10	−0.2 (4)
C2—C1—C4—C5	−179.39 (17)	C8—C9—C10—C11	0.0 (4)
C1—C4—C5—O5	11.1 (3)	C9—C10—C11—N1	0.1 (4)
C1—C4—C5—O4	−169.0 (2)	N2—C7—N1—C11	179.6 (2)
O1—C1—C6—O7	21.0 (2)	C8—C7—N1—C11	−0.5 (3)
C4—C1—C6—O7	142.6 (2)	C10—C11—N1—C7	0.2 (4)

### 2-Aminopyridin-1-ium 3-carboxy-2-carboxymethyl-2-hydroxypropanoate (I) Hydrogen-bond geometry (Å, º)

**Table:**

*D*—H···*A*	*D*—H	H···*A*	*D*···*A*	*D*—H···*A*
O1—H1···O6^i^	0.82	1.86	2.681 (4)	177
N1—H1*A*···O1^ii^	0.86	2.09	2.895 (4)	156
N2—H2*C*···O1^ii^	0.86	2.34	3.076 (5)	144
N2—H2*D*···O2	0.86	2.09	2.935 (5)	168
O3—H3···O7^i^	0.82	1.75	2.547 (4)	164
O4—H4···O6^iii^	0.82	1.82	2.601 (4)	158
C9—H9···O3^iv^	0.93	2.57	3.351 (5)	142

Symmetry codes: (i) −*x*+1/2, *y*+1/2, *z*; (ii) *x*+1/2, *y*, −*z*+1/2; (iii) *x*−1/2, −*y*+1/2, −*z*+1; (iv) −*x*+1/2, *y*−1/2, *z*.

### Tris(2-aminopyridin-1-ium) 2-hydroxypropane-1,2,3-tricarboxylate (II) Crystal data

**Table:**

3C_5_H_7_N_2_^+^·C_6_H_5_O_7_^3^^−^	*F*(000) = 1000
*M**_r_* = 474.48	*D*_x_ = 1.368 Mg m^−^^3^
Monoclinic, *P*2_1_/*n*	Mo *K*α radiation, λ = 0.71073 Å
*a* = 10.0297 (17) Å	Cell parameters from 132 reflections
*b* = 10.6564 (14) Å	θ = 3.1–27.5°
*c* = 21.986 (4) Å	µ = 0.11 mm^−^^1^
β = 101.426 (9)°	*T* = 296 K
*V* = 2303.3 (7) Å^3^	Prism, colourless
*Z* = 4	0.22 × 0.19 × 0.17 mm

### Tris(2-aminopyridin-1-ium) 2-hydroxypropane-1,2,3-tricarboxylate (II) Data collection

**Table:**

Bruker APEXII diffractometer	5242 independent reflections
Radiation source: sealed X-ray tube	3779 reflections with *I* > 2σ(*I*)
Graphite monochromator	*R*_int_ = 0.056
phi and φ scans	θ_max_ = 27.5°, θ_min_ = 3.1°
Absorption correction: multi-scan (SADABS; Bruker, 2009)	*h* = −13→12
*T*_min_ = 0.977, *T*_max_ = 0.982	*k* = −13→13
13120 measured reflections	*l* = −28→16

### Tris(2-aminopyridin-1-ium) 2-hydroxypropane-1,2,3-tricarboxylate (II) Refinement

**Table:**

Refinement on *F*^2^	Primary atom site location: structure-invariant direct methods
Least-squares matrix: full	Secondary atom site location: difference Fourier map
*R*[*F*^2^ > 2σ(*F*^2^)] = 0.052	Hydrogen site location: mixed
*wR*(*F*^2^) = 0.149	H atoms treated by a mixture of independent and constrained refinement
*S* = 1.05	*w* = 1/[σ^2^(*F*_o_^2^) + (0.0673*P*)^2^ + 0.409*P*] where *P* = (*F*_o_^2^ + 2*F*_c_^2^)/3
5242 reflections	(Δ/σ)_max_ < 0.001
311 parameters	Δρ_max_ = 0.27 e Å^−^^3^
0 restraints	Δρ_min_ = −0.21 e Å^−^^3^

### Tris(2-aminopyridin-1-ium) 2-hydroxypropane-1,2,3-tricarboxylate (II) Special details

**Table:**

Geometry. All esds (except the esd in the dihedral angle between two l.s. planes) are estimated using the full covariance matrix. The cell esds are taken into account individually in the estimation of esds in distances, angles and torsion angles; correlations between esds in cell parameters are only used when they are defined by crystal symmetry. An approximate (isotropic) treatment of cell esds is used for estimating esds involving l.s. planes.

### Tris(2-aminopyridin-1-ium) 2-hydroxypropane-1,2,3-tricarboxylate (II) Fractional atomic coordinates and isotropic or equivalent isotropic displacement parameters (Å^2^)

**Table:**

	*x*	*y*	*z*	*U*_iso_*/*U*_eq_	
O1	0.40837 (15)	0.54865 (11)	0.08093 (6)	0.0423 (3)	
H1O	0.489 (3)	0.506 (3)	0.0879 (12)	0.071 (8)*	
O2	0.52383 (14)	0.18918 (12)	0.03079 (6)	0.0466 (3)	
O3	0.59153 (14)	0.36464 (13)	0.08316 (7)	0.0527 (4)	
O4	0.00785 (17)	0.50955 (14)	0.11124 (8)	0.0651 (5)	
O5	0.13362 (17)	0.67594 (14)	0.10506 (9)	0.0661 (5)	
O6	0.25602 (17)	0.28972 (13)	0.14580 (6)	0.0542 (4)	
O7	0.36839 (14)	0.45764 (12)	0.18962 (6)	0.0449 (3)	
C1	0.31580 (17)	0.44500 (15)	0.07766 (7)	0.0322 (4)	
C2	0.35607 (19)	0.34310 (16)	0.03557 (8)	0.0377 (4)	
H2A	0.295896	0.271947	0.035888	0.045*	
H2B	0.339938	0.375355	−0.006509	0.045*	
C3	0.50118 (19)	0.29614 (16)	0.05164 (8)	0.0368 (4)	
C4	0.17245 (19)	0.49234 (18)	0.04858 (8)	0.0410 (4)	
H4A	0.179056	0.544641	0.013183	0.049*	
H4B	0.116466	0.420505	0.033149	0.049*	
C5	0.10047 (18)	0.56668 (17)	0.09152 (8)	0.0393 (4)	
C6	0.31417 (17)	0.39294 (15)	0.14353 (8)	0.0334 (4)	
N1	0.14007 (18)	0.20220 (15)	0.24858 (8)	0.0510 (4)	
H1A	0.137464	0.124190	0.258293	0.061*	
H1B	0.156634	0.223370	0.213063	0.061*	
N2	0.09288 (17)	0.25593 (15)	0.34395 (7)	0.0458 (4)	
H2	0.093048	0.175597	0.351991	0.055*	
C7	0.11865 (18)	0.29025 (17)	0.28856 (8)	0.0381 (4)	
C8	0.1228 (2)	0.42056 (17)	0.27643 (9)	0.0451 (4)	
H8	0.138649	0.448570	0.238434	0.054*	
C9	0.1037 (2)	0.50434 (19)	0.32022 (11)	0.0535 (5)	
H9	0.108433	0.589807	0.312368	0.064*	
C10	0.0768 (2)	0.4638 (2)	0.37722 (11)	0.0603 (6)	
H10	0.062386	0.521166	0.407137	0.072*	
C11	0.0723 (2)	0.3385 (2)	0.38754 (10)	0.0558 (5)	
H11	0.054862	0.309350	0.425062	0.067*	
N3	0.8662 (2)	0.27826 (16)	0.08313 (9)	0.0557 (5)	
H3A	0.785814	0.310176	0.076107	0.067*	
H3B	0.936026	0.325756	0.094377	0.067*	
N4	0.77000 (16)	0.08491 (14)	0.05893 (8)	0.0430 (4)	
H4	0.692251	0.121911	0.051585	0.052*	
C12	0.8824 (2)	0.15464 (18)	0.07656 (8)	0.0421 (4)	
C13	1.0095 (2)	0.0933 (2)	0.08716 (9)	0.0504 (5)	
H13	1.089615	0.138975	0.098593	0.060*	
C14	1.0135 (2)	−0.0336 (2)	0.08043 (10)	0.0558 (5)	
H14	1.097062	−0.074390	0.087536	0.067*	
C15	0.8939 (2)	−0.1037 (2)	0.06300 (11)	0.0551 (5)	
H15	0.897109	−0.190487	0.058988	0.066*	
C16	0.7740 (2)	−0.04213 (18)	0.05220 (10)	0.0485 (5)	
H16	0.693459	−0.086724	0.040050	0.058*	
N5	0.62921 (16)	0.12553 (15)	0.31762 (7)	0.0441 (4)	
H5	0.587297	0.102488	0.346297	0.053*	
N6	0.46530 (18)	0.27662 (17)	0.28874 (8)	0.0514 (4)	
H6A	0.426137	0.251756	0.318032	0.062*	
H6B	0.431257	0.337666	0.265102	0.062*	
C17	0.57809 (19)	0.22085 (17)	0.28005 (8)	0.0393 (4)	
C18	0.6471 (2)	0.2548 (2)	0.23248 (10)	0.0499 (5)	
H18	0.613328	0.318660	0.204882	0.060*	
C19	0.7631 (3)	0.1936 (2)	0.22721 (12)	0.0640 (6)	
H19	0.808573	0.216020	0.195845	0.077*	
C20	0.8150 (2)	0.0974 (2)	0.26828 (13)	0.0686 (7)	
H20	0.895861	0.056931	0.265660	0.082*	
C21	0.7437 (2)	0.0652 (2)	0.31185 (11)	0.0580 (6)	
H21	0.774715	−0.000586	0.338719	0.070*	

### Tris(2-aminopyridin-1-ium) 2-hydroxypropane-1,2,3-tricarboxylate (II) Atomic displacement parameters (Å^2^)

**Table:**

	*U*^11^	*U*^22^	*U*^33^	*U*^12^	*U*^13^	*U*^23^
O1	0.0546 (8)	0.0294 (6)	0.0474 (7)	−0.0046 (6)	0.0211 (6)	−0.0017 (5)
O2	0.0522 (8)	0.0363 (7)	0.0538 (8)	0.0038 (6)	0.0162 (6)	−0.0095 (6)
O3	0.0496 (8)	0.0504 (8)	0.0574 (8)	0.0004 (6)	0.0084 (6)	−0.0184 (7)
O4	0.0731 (10)	0.0523 (8)	0.0824 (11)	−0.0157 (8)	0.0453 (9)	−0.0252 (8)
O5	0.0646 (10)	0.0438 (8)	0.0986 (13)	−0.0062 (7)	0.0371 (9)	−0.0227 (8)
O6	0.0796 (10)	0.0415 (7)	0.0443 (8)	−0.0156 (7)	0.0187 (7)	0.0038 (6)
O7	0.0567 (8)	0.0452 (7)	0.0320 (6)	0.0011 (6)	0.0070 (6)	−0.0052 (6)
C1	0.0407 (9)	0.0277 (7)	0.0297 (8)	0.0003 (7)	0.0104 (7)	−0.0011 (6)
C2	0.0463 (10)	0.0357 (8)	0.0328 (8)	0.0013 (7)	0.0118 (7)	−0.0069 (7)
C3	0.0493 (10)	0.0334 (8)	0.0312 (8)	−0.0005 (7)	0.0162 (7)	−0.0022 (7)
C4	0.0486 (10)	0.0422 (9)	0.0321 (8)	0.0086 (8)	0.0074 (8)	0.0000 (7)
C5	0.0413 (9)	0.0377 (9)	0.0371 (9)	0.0069 (8)	0.0035 (7)	−0.0036 (7)
C6	0.0391 (9)	0.0303 (8)	0.0325 (8)	0.0048 (7)	0.0111 (7)	0.0001 (7)
N1	0.0642 (11)	0.0406 (8)	0.0532 (10)	−0.0002 (8)	0.0238 (8)	0.0016 (7)
N2	0.0544 (10)	0.0389 (8)	0.0478 (9)	0.0046 (7)	0.0187 (7)	0.0134 (7)
C7	0.0362 (9)	0.0380 (9)	0.0420 (9)	0.0023 (7)	0.0123 (7)	0.0074 (7)
C8	0.0523 (11)	0.0387 (9)	0.0466 (10)	0.0012 (8)	0.0154 (9)	0.0131 (8)
C9	0.0613 (13)	0.0362 (9)	0.0639 (13)	0.0037 (9)	0.0147 (10)	0.0054 (9)
C10	0.0729 (15)	0.0564 (13)	0.0546 (12)	0.0095 (11)	0.0200 (11)	−0.0082 (11)
C11	0.0659 (13)	0.0634 (13)	0.0431 (11)	0.0075 (11)	0.0228 (10)	0.0079 (10)
N3	0.0647 (11)	0.0406 (9)	0.0642 (11)	−0.0118 (8)	0.0184 (9)	−0.0069 (8)
N4	0.0404 (8)	0.0371 (8)	0.0514 (9)	0.0001 (7)	0.0089 (7)	−0.0042 (7)
C12	0.0513 (11)	0.0418 (9)	0.0351 (9)	−0.0075 (8)	0.0129 (8)	−0.0021 (8)
C13	0.0447 (11)	0.0613 (12)	0.0446 (11)	−0.0089 (9)	0.0077 (9)	−0.0067 (9)
C14	0.0462 (11)	0.0624 (13)	0.0577 (13)	0.0107 (10)	0.0076 (9)	−0.0019 (11)
C15	0.0558 (12)	0.0418 (10)	0.0655 (14)	0.0063 (9)	0.0071 (10)	−0.0038 (10)
C16	0.0475 (11)	0.0384 (9)	0.0585 (12)	−0.0054 (8)	0.0080 (9)	−0.0078 (9)
N5	0.0424 (8)	0.0461 (9)	0.0445 (8)	0.0010 (7)	0.0099 (7)	0.0143 (7)
N6	0.0518 (10)	0.0561 (10)	0.0475 (9)	0.0107 (8)	0.0129 (8)	0.0170 (8)
C17	0.0409 (9)	0.0383 (9)	0.0374 (9)	−0.0019 (7)	0.0041 (7)	0.0047 (7)
C18	0.0559 (12)	0.0475 (10)	0.0476 (11)	0.0002 (9)	0.0130 (9)	0.0140 (9)
C19	0.0646 (14)	0.0676 (15)	0.0682 (15)	0.0001 (12)	0.0331 (12)	0.0167 (12)
C20	0.0558 (13)	0.0658 (14)	0.0919 (18)	0.0140 (11)	0.0331 (13)	0.0234 (14)
C21	0.0491 (12)	0.0547 (12)	0.0709 (14)	0.0092 (10)	0.0136 (10)	0.0230 (11)

### Tris(2-aminopyridin-1-ium) 2-hydroxypropane-1,2,3-tricarboxylate (II) Geometric parameters (Å, º)

**Table:**

O1—C1	1.435 (2)	C11—H11	0.9300
O1—H1O	0.92 (3)	N3—C12	1.339 (3)
O2—C3	1.266 (2)	N3—H3A	0.8600
O3—C3	1.259 (2)	N3—H3B	0.8601
O4—C5	1.257 (2)	N4—C12	1.342 (2)
O5—C5	1.231 (2)	N4—C16	1.363 (2)
O6—C6	1.251 (2)	N4—H4	0.8601
O7—C6	1.257 (2)	C12—C13	1.410 (3)
C1—C2	1.532 (2)	C13—C14	1.362 (3)
C1—C4	1.538 (2)	C13—H13	0.9300
C1—C6	1.554 (2)	C14—C15	1.400 (3)
C2—C3	1.513 (3)	C14—H14	0.9300
C2—H2A	0.9700	C15—C16	1.349 (3)
C2—H2B	0.9700	C15—H15	0.9300
C4—C5	1.520 (2)	C16—H16	0.9300
C4—H4A	0.9700	N5—C21	1.344 (3)
C4—H4B	0.9700	N5—C17	1.345 (2)
N1—C7	1.332 (3)	N5—H5	0.8600
N1—H1A	0.8600	N6—C17	1.325 (3)
N1—H1B	0.8601	N6—H6A	0.8599
N2—C7	1.345 (2)	N6—H6B	0.8600
N2—C11	1.347 (3)	C17—C18	1.411 (3)
N2—H2	0.8740	C18—C19	1.359 (3)
C7—C8	1.416 (2)	C18—H18	0.9300
C8—C9	1.354 (3)	C19—C20	1.397 (3)
C8—H8	0.9300	C19—H19	0.9300
C9—C10	1.401 (3)	C20—C21	1.348 (3)
C9—H9	0.9300	C20—H20	0.9300
C10—C11	1.356 (3)	C21—H21	0.9300
C10—H10	0.9300		
			
C1—O1—H1O	99.9 (17)	N2—C11—C10	120.6 (2)
O1—C1—C2	109.29 (14)	N2—C11—H11	119.7
O1—C1—C4	108.09 (14)	C10—C11—H11	119.7
C2—C1—C4	108.54 (13)	C12—N3—H3A	120.0
O1—C1—C6	110.73 (13)	C12—N3—H3B	120.0
C2—C1—C6	111.26 (13)	H3A—N3—H3B	120.0
C4—C1—C6	108.84 (14)	C12—N4—C16	122.68 (17)
C3—C2—C1	116.74 (14)	C12—N4—H4	118.6
C3—C2—H2A	108.1	C16—N4—H4	118.7
C1—C2—H2A	108.1	N3—C12—N4	117.58 (19)
C3—C2—H2B	108.1	N3—C12—C13	124.28 (19)
C1—C2—H2B	108.1	N4—C12—C13	118.14 (18)
H2A—C2—H2B	107.3	C14—C13—C12	119.12 (19)
O3—C3—O2	124.07 (17)	C14—C13—H13	120.4
O3—C3—C2	119.39 (15)	C12—C13—H13	120.4
O2—C3—C2	116.53 (16)	C13—C14—C15	121.2 (2)
C5—C4—C1	115.63 (14)	C13—C14—H14	119.4
C5—C4—H4A	108.4	C15—C14—H14	119.4
C1—C4—H4A	108.4	C16—C15—C14	118.25 (19)
C5—C4—H4B	108.4	C16—C15—H15	120.9
C1—C4—H4B	108.4	C14—C15—H15	120.9
H4A—C4—H4B	107.4	C15—C16—N4	120.57 (19)
O5—C5—O4	123.87 (18)	C15—C16—H16	119.7
O5—C5—C4	120.28 (18)	N4—C16—H16	119.7
O4—C5—C4	115.85 (16)	C21—N5—C17	122.15 (18)
O6—C6—O7	125.52 (17)	C21—N5—H5	118.9
O6—C6—C1	116.25 (15)	C17—N5—H5	118.9
O7—C6—C1	118.22 (15)	C17—N6—H6A	120.0
C7—N1—H1A	120.0	C17—N6—H6B	120.0
C7—N1—H1B	120.0	H6A—N6—H6B	120.0
H1A—N1—H1B	120.0	N6—C17—N5	118.87 (17)
C7—N2—C11	123.42 (17)	N6—C17—C18	123.38 (17)
C7—N2—H2	117.2	N5—C17—C18	117.74 (18)
C11—N2—H2	119.4	C19—C18—C17	119.63 (19)
N1—C7—N2	119.41 (17)	C19—C18—H18	120.2
N1—C7—C8	123.51 (17)	C17—C18—H18	120.2
N2—C7—C8	117.08 (17)	C18—C19—C20	120.8 (2)
C9—C8—C7	119.94 (18)	C18—C19—H19	119.6
C9—C8—H8	120.0	C20—C19—H19	119.6
C7—C8—H8	120.0	C21—C20—C19	117.6 (2)
C8—C9—C10	120.79 (19)	C21—C20—H20	121.2
C8—C9—H9	119.6	C19—C20—H20	121.2
C10—C9—H9	119.6	N5—C21—C20	122.0 (2)
C11—C10—C9	118.1 (2)	N5—C21—H21	119.0
C11—C10—H10	120.9	C20—C21—H21	119.0
C9—C10—H10	120.9		
			
O1—C1—C2—C3	54.58 (19)	C7—C8—C9—C10	−1.4 (3)
C4—C1—C2—C3	172.25 (15)	C8—C9—C10—C11	0.9 (4)
C6—C1—C2—C3	−68.0 (2)	C7—N2—C11—C10	0.0 (3)
C1—C2—C3—O3	−22.8 (2)	C9—C10—C11—N2	−0.2 (4)
C1—C2—C3—O2	158.57 (16)	C16—N4—C12—N3	−178.41 (19)
O1—C1—C4—C5	−77.58 (19)	C16—N4—C12—C13	1.4 (3)
C2—C1—C4—C5	163.99 (15)	N3—C12—C13—C14	178.4 (2)
C6—C1—C4—C5	42.8 (2)	N4—C12—C13—C14	−1.3 (3)
C1—C4—C5—O5	75.6 (2)	C12—C13—C14—C15	0.3 (3)
C1—C4—C5—O4	−104.5 (2)	C13—C14—C15—C16	0.8 (4)
O1—C1—C6—O6	−167.93 (15)	C14—C15—C16—N4	−0.8 (3)
C2—C1—C6—O6	−46.2 (2)	C12—N4—C16—C15	−0.3 (3)
C4—C1—C6—O6	73.39 (19)	C21—N5—C17—N6	−179.5 (2)
O1—C1—C6—O7	13.2 (2)	C21—N5—C17—C18	1.6 (3)
C2—C1—C6—O7	135.00 (16)	N6—C17—C18—C19	179.3 (2)
C4—C1—C6—O7	−105.44 (17)	N5—C17—C18—C19	−1.8 (3)
C11—N2—C7—N1	178.85 (19)	C17—C18—C19—C20	0.0 (4)
C11—N2—C7—C8	−0.5 (3)	C18—C19—C20—C21	2.0 (4)
N1—C7—C8—C9	−178.14 (19)	C17—N5—C21—C20	0.5 (4)
N2—C7—C8—C9	1.2 (3)	C19—C20—C21—N5	−2.3 (4)

### Tris(2-aminopyridin-1-ium) 2-hydroxypropane-1,2,3-tricarboxylate (II) Hydrogen-bond geometry (Å, º)

**Table:**

*D*—H···*A*	*D*—H	H···*A*	*D*···*A*	*D*—H···*A*
O1—H1*O*···O3	0.91 (3)	1.84 (3)	2.681 (2)	152 (3)
N3—H3*A*···O3	0.86	2.07	2.905 (3)	164
N4—H4···O2	0.86	1.81	2.666 (2)	175
N1—H1*B*···O6	0.86	2.07	2.893 (2)	161
N6—H6*B*···O7	0.86	2.09	2.928 (2)	164
N1—H1*A*···O7^i^	0.86	2.12	2.948 (2)	162
N2—H2···O1^i^	0.86	2.00	2.760 (2)	144
N2—H2···O7^i^	0.86	2.55	3.304 (2)	144
C9—H9···O6^ii^	0.93	2.60	3.372 (3)	141
C10—H10···O2^ii^	0.93	2.51	3.419 (3)	167
C11—H11···O2^iii^	0.93	2.41	3.294 (3)	160
N3—H3*B*···O4^iv^	0.86	2.09	2.851 (2)	146
C13—H13···O6^iv^	0.93	2.40	3.301 (3)	163
N5—H5···O4^i^	0.86	1.77	2.591 (2)	160
N6—H6*A*···O5^i^	0.86	2.07	2.916 (3)	169
C20—H20···O7^v^	0.93	2.60	3.463 (3)	155
C21—H21···O3^v^	0.93	2.43	3.334 (3)	164

Symmetry codes: (i) −*x*+1/2, *y*−1/2, −*z*+1/2; (ii) −*x*+1/2, *y*+1/2, −*z*+1/2; (iii) *x*−1/2, −*y*+1/2, *z*+1/2; (iv) *x*+1, *y*, *z*; (v) −*x*+3/2, *y*−1/2, −*z*+1/2.
